# Synthesis, Purification and Characterization of Polymerizable Multifunctional Quaternary Ammonium Compounds

**DOI:** 10.3390/molecules24081464

**Published:** 2019-04-13

**Authors:** Ugochukwu C. Okeke, Chad R. Snyder, Stanislav A. Frukhtbeyn

**Affiliations:** 1 Volpe Research Center, American Dental Association Foundation, 100 Bureau Dr., Stop 8546, Gaithersburg, MD 20899, USA; 2 Materials Science and Engineering Division, National Institute of Standards and Technology, 100 Bureau Dr., Stop 8546, Gaithersburg, MD 20899, USA; chadsnyd@nist.gov

**Keywords:** quaternary ammonium salts, antimicrobial monomers, antibacterial dental materials, tooth caries inhibition, Menschutkin reaction, copolymers, composites, resins

## Abstract

Methacrylate analogs of quaternary ammonium salts functionalized with carboxylic (AMadh1 68.8% yield, AMadh2 53.2% yield) and methoxysilane (AMsil1 94.8% yield, AMsil2 36.0% yield) groups were synthesized via Menschutkin reaction. Fourier-transform infrared spectroscopy (FTIR), nuclear magnetic resonance spectroscopy (^1^H, ^13^C and 2D ^1^H-^13^C heteronuclear single quantum coherence (HSQC) NMR), mass spectrometry, thermogravimetric analysis (TGA) and differential scanning calorimetry (DSC) were utilized to validate structures and characterize thermal properties of the novel multifunctional quaternary ammonium salts synthesized. The potential adhesive, coupling and antimicrobial properties of these multifunctional monomers encourage their further comprehensive evaluation in conventional and experimental copolymers and composites.

## 1. Introduction

Quaternary ammonium (QA) salts are highly stable compounds [1], known for their phase transfer catalytic [2,3], and germicidal properties [4,5]. Their antimicrobial (AM) potential makes QAs particularly useful in materials applications where AM activity is highly desired, such as dental materials. There has been increased interest in the design of QA compounds with polymerizable functional groups as they may provide materials with desired AM properties. Past studies on the introduction of AM agents, such as chlorhexidine, antibiotics, zinc, silver, fluoride and iodide [6,7,8,9], into such materials have only resulted in short-term AM effect particularly in dental materials upon prolonged exposure to the intra-oral environment. To resolve the issue of unsustainable AM effect, many efforts have been devoted to the design and synthesis of polymerizable QA compounds [8,10,11,12,13] Their AM action has been reported to depend on the type of counter ion [10], pendant active groups [6] and length of the alkyl chains [7]. There has been a desire to design and synthesize QAs with carboxylate and silane functional groups. Carboxylate functionality has been widely used in dental materials to facilitate adhesion to dentin and/or enamel [14,15,16]. On the other hand, organosilanes are traditionally utilized as good coupling agents with both siliceous fillers and the polymeric matrix [17] In addition, it has been demonstrated that QAs with hydrophobic alkyl chains provide AM function that increases with the chain length [18,19]. Increased chain length also increases the hydrophobicity of the resins/composites thus decreasing the potential for excessive water uptake [20]. The objective of this work was to incorporate QA AM functionality with either carboxylate (AMadh series) or organosilane (AMsil series) into methacrylate-based monomers with varied alkyl chain length. Complex structural characterization of these materials was performed to validate their structures and the synthetic route utilized. Evaluation of their thermal properties was performed to compare their phase transition behavior relative to other dental monomers.

## 2. Results and Discussion

**Scheme 1:**
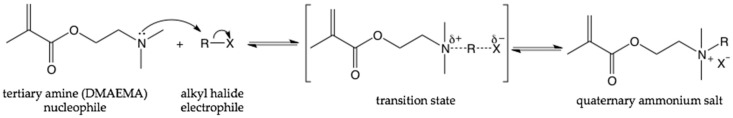
S_N_2 mechanism of quaternary ammonium salt formation.

Quaternary ammonium salts were synthesized by reacting a tertiary amine with four commercially available alkyl halides, containing carboxylic (AMadh series) or organosilane (AMsil series) functional groups. The reaction between a neutral amine and an alkyl halide producing two ions of opposite sign, also known as a Menschutkin reaction [21,22], is a bimolecular nucleophilic substitution (S_N_2) reaction (Scheme 1). This reaction occurs at an aliphatic carbon with a stable, electronegative leaving group (halide). The breaking of the carbon-halide bond and the formation of the new nitrogen-carbon bond occurs simultaneously during the transition state. In solutions, final products may exist as separate ions, ion pairs or solid salts. Reversibility of the Menschutkin reactions in solutions has been well established [23,24].

Typically, Menschutkin reactions are conducted in polar solvents, which stabilize the transition state, increasing the rate of reaction. Density functional theory (DFT) calculations [25] show progressive lengthening of carbon-halide and shortening of carbon-nitrogen bonds of the transition state moiety with an increase of dielectric constant of the solvent used. Our choice of a solvent (chloroform) with a relatively low dielectric constant (ε = 4.81) was dictated by the presence of carboxylic and methoxy-silane groups in one of the reactants. Dissociation of the carboxylic acid proton would be minimized, relative to a more polar solvent, which may participate in an unwanted acid-base interaction with a tertiary amine. Formation of a charged quaternary ammonium salt with highly polar adducts in chloroform can also lead to its precipitation, which may significantly simplify its purification from the reacting mixture. Direct precipitation of the reaction product was only observed in the case of AMadh2 (53.2% yield). Other products remained dissolved, possibly due to solvent hydrogen bonding to their longer alkyl chains. Additions of low polarity solvents (hexane, diethyl ether) led to the precipitation of purified products (AMadh1 68.8%, AMsil1 94.8% and AMsil2 36.0% yields). In polar aprotic solvents, iodide is a significantly better leaving group than bromide, which explains a 2.6-fold increase in AMsil1 product yield (relative to AMsil2).

FT-IR spectra (Supplementary Figures S1 and S2) of synthesized compounds were consistent with their proposed structures. Absorption peaks (3000 to 3020) cm^−1^ (sp2 C-H stretch), (2916 to 2943) cm^−1^ (sp3 C-H stretch), (1716 to 1721) cm^−1^ (C=O stretch), (1636 to 1641) cm^−1^ (C=C stretch) were observed in all products. Additionally, AMsil series products exhibited (1076 to 1082) cm^−1^ peaks corresponding to Si-O-C asymmetric stretch [26,27,28].

**Figure 1:**
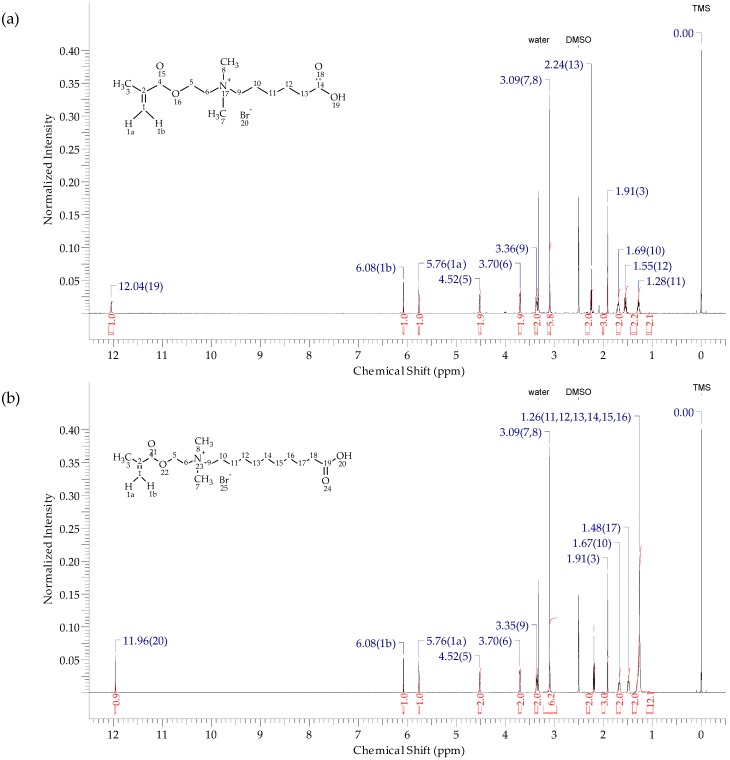
^1^H-NMR spectra of AMadh1 (**a**) and AMadh2 (**b**) products.

NMR spectra of AMadh series compounds and their structures are shown in Figure 1 (^1^H-NMR) and Supplementary Figures S4, S6 and S7 (^13^C-NMR, HSQC). Distinctive signals assigned to the carboxylic acid proton were observed at a chemical shift of 12.04 ppm (AMadh1) and 11.96 ppm (AMadh2). The two vinyl protons were observed at 6.08 (trans-) and 5.76 (cis-) ppm, confirming successful inhibition of methacrylate self-polymerization during the synthesis. Both AMadh compounds exhibit a singlet at 3.09 ppm, which was assigned to the methyl groups (positions 7, 8) directly attached to the quaternary amine (–N^+^(CH_3_)_2_–), which corresponded to 50.4 ppm chemical shift in their ^13^C-NMR spectra. Chemical shifts of 4.52 (58.0, 58.1) and 3.70 (61.6) ppm were assigned to protons (carbons) on the methylene bridge (–O–CH_2_–CH_2_–N^+^(CH_3_)_2_–, positions 5 and 6 respectively). Significant downfield shift of these signals in comparison to the ones present in their tertiary amine precursor DMAEMA (^1^H/^13^C chemical shifts: 2.18/45.3 ppm (positions 7, 8) and 2.52/57.2 ppm (position 6), Supplementary Figure S3), is due to the de-shielding effect of a positively charged nitrogen center and further confirms the success of the quaternization reaction. Complete NMR structural assignment of AMadh compounds is given in Table 1 and Table 2.

**Figure 2:**
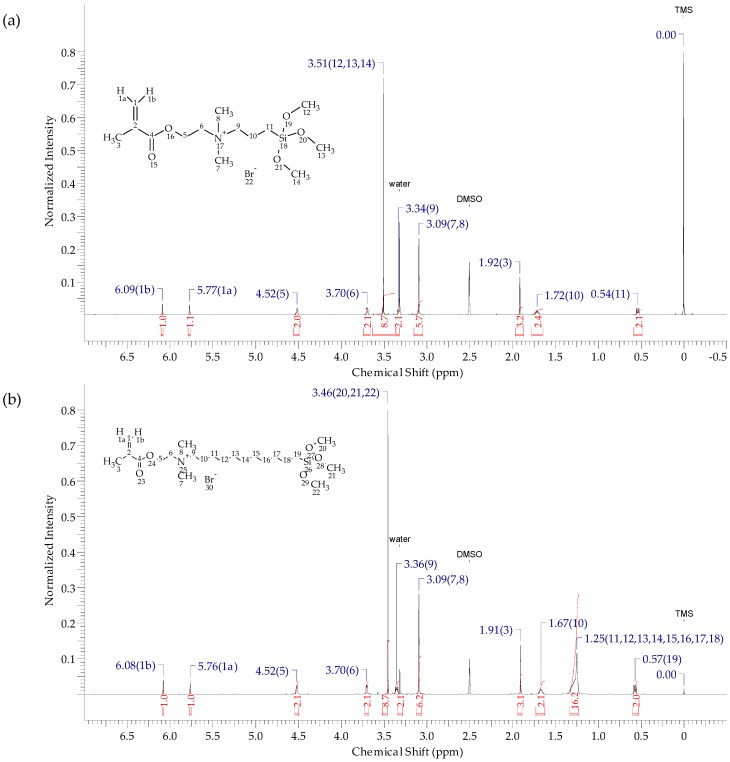
^1^H-NMR spectra of AMsil1 (**a**) and AMsil2 (**b**) products.

NMR spectra of AMsil series compounds shown in Figure 2 (^1^H-NMR) and Supplementary Figures S5, S8, and S9 (^13^C, HSQC) also confirm successful quaternization of the tertiary amine reactant. It must be noted that methoxy groups (Si–(O–CH_3_)_3_, assigned to ^1^H/^13^C chemical shifts of 3.51/50.1 (AMsil1), 3.46/49.9 ppm (AMsil2); positions 20, 21, 22), which are highly prone to hydrolysis in the presence of moisture, were successfully protected by performing this synthesis in chloroform. Complete NMR structural assignment of AMsil compounds is given in Table 3 and Table 4.

Molecular ions (M^+^) of AMadh2 (*m*/*z* = 342.4, 343.4, 344.4), AMsil1 (*m*/*z* = 320.3, 321.3, 322.2) and AMsil2 (*m*/*z* = 432.4, 433.4, 434.4) were directly observed during mass spectrometric analysis of the synthesized compounds (Supplementary Figures S10–S13). All compounds showed high abundance of C_6_H_9_O_2_^•^ fragments (*m*/*z* = 113.1), typically seen in mass spectra of mono- and di-methacrylates. Collectively, FTIR, NMR and MS analysis of synthesized AMadh and AMsil series compounds confirmed that their structures were as designed.

Thermogravimetric analysis of synthesized monomers (Supplementary Figure S14) revealed that they were stable at physiologically relevant temperatures. Thermal degradation onset was observed at ≈150 °C for all compounds. Minor sample mass changes at or below 100 °C can be associated with water loss of 1% to 2% mass fraction for AMsil monomers and 3% to 4% mass fraction for AMadh2 monomer.

**Figure 3:**
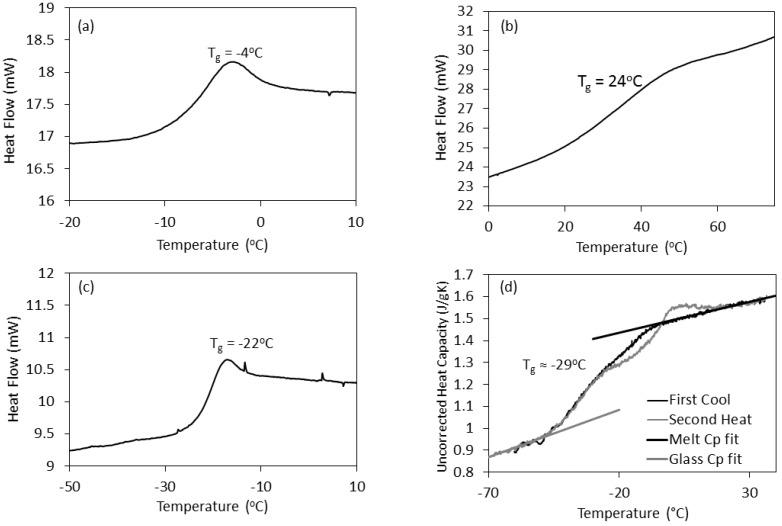
Typical DSC thermograms of the glass phase transitions of the first heating ramp (10 °C/min) for (**a**) AMadh1,(**b**) AMadh2, (**c**) AMsil1 and first cool/second heat curves of (**d**) AMsil2.

DSC thermograms of the first heating ramp revealed glassy phase transitions for three of the four synthesized monomers (Figure 3). Glass transition temperatures (T_g_) and the corresponding changes in specific heat capacity (ΔC_p_) for AMadh1, AMadh2 and AMsil1 were determined to be approximately −4, 24, and −22 °C and 0.52, 0.38, and 0.59 J/(g K), respectively. Further heating, revealed exothermic cold crystallization peaks at ≈86 and ≈48 °C with a change in enthalpy (ΔH) of ≈−60 and −41 J/g for AMadh1 and AMsil1, respectively (Supplementary Figures S15–S18). Endothermic melting phase transition peaks were observed at ≈125 °C (ΔH ≈ 122 J/g), ≈132 °C (ΔH ≈ 167 J/g) and ≈93 °C (ΔH ≈ 39 J/g) for AMadh1, AMadh2 and AMsil1, respectively. Analysis of scans of the first cooling ramp suggests that AMadh1 and AMsil1 monomers are slow crystallizers because they show a cold crystallization peak on first heating with no corresponding crystallization peak on subsequent cooling from the melt. However, AMadh2 and AMsil2 presented crystallization peaks upon cooling at ≈83 °C (ΔH ≈ −133 J/g) and ≈13 °C (ΔH ≈ −3 J/g). Melting temperatures of monomers obtained from the thermograms of the second heating ramp were in good agreement with values observed in the first ramp. AMsil2 was not amenable to the typical heating and cooling analysis, possibly due to residual moisture; therefore, additional measurements were performed on a DSC (TA Instruments Q1000) with improved baseline linearity. The heating data showed multiple inflections (Figure 3d), which after matching with cooling heat capacity data could be attributed to a cold crystallization at ≈11 °C followed by melting at ≈2 °C. Because the base cooling temperature of the DSC was −90 °C and presence of spikes in the cooling data (likely due to mechanical effects in the pan) the cooling data alone was insufficient to obtain a reasonable estimate for the crystal/glassy C_p_ line. To eliminate the mechanical effects, two sets of cooling data were obtained and averaged, while excluding the regions around the spikes in the baseline. The overlap in C_p_ between heating and cooling at low temperatures gave sufficient confidence that the heating data could be used for the crystal/glassy C_p_ line and the overlap at high temperatures for both gave confidence that the cooling trace could be used to estimate the liquid/melt C_p_ line. Both crystal/glassy and liquid/melt lines were determined with a linear least squares fit to a straight line and the glass transition temperature of AMsil2 was estimated by the intersection of the cooling data at (1/2)ΔC_p,glass-melt_ as ≈−29 °C (ΔC_p_ ≈ 0.36 J/(g K)).

**Figure 4:**
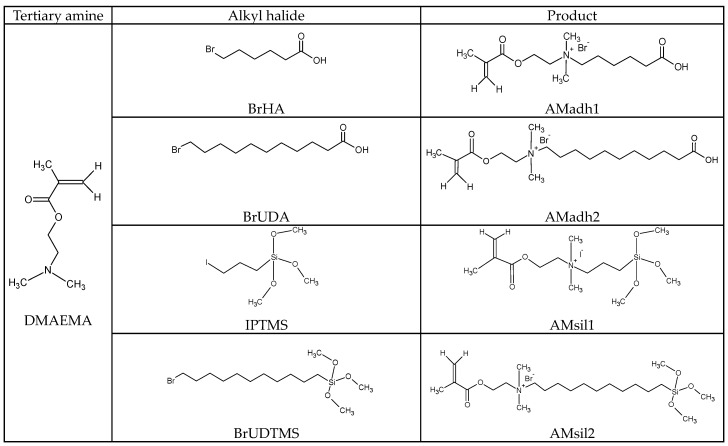
Reactants and corresponding quaternary ammonium monomer products.

The measured glass transition temperatures of the synthesized methacrylate monomer salts (Figure 4) are generally in line with monomeric T_g_’s of base monomers commonly utilized in dental resins. Differential scanning calorimetry of urethane dimethacrylate (UDMA) and bisphenol A glycidyl methacrylate (BisGMA) established their monomeric T_g_’s to be −38 °C and −10 °C, respectively. [29] Incorporation of these QA methacrylate monomer salts into comonomer mixtures based on BisGMA or UDMA will be expected to contribute to the glass transition temperature of the mixture in an additive fashion. However, structural differences between the QA monomer salts and base monomers may give rise to polymerization-induced phase separation in certain formulations. These types of partially structurally and thermodynamically compatible comonomer formulations have been reported to form heterogeneous polymer networks that exhibit reduced polymerization shrinkage and stress development [30,31,32]. We are currently evaluating these effects of QA monomers and will report detailed findings in future publications.

## 3. Materials and Methods

All solvents and reagents were procured from commercial sources and were used without any further purification. 2-(dimethylamino)ethyl methacrylate (DMAEMA), 6-bromohexanoic acid (BrHA), 11-bromoundecanoic acid (BrUDA), butylated hydroxytoluene (BHT), and (3-iodopropyl)trimethoxysilane (IPTMS) were purchased from Sigma (St. Louis, MO, USA). Chloroform was purchased from Acros Organics (Geel, Belgium) and (11-bromoundecyl)trimethoxysilane (BrUDTMS) was from Gelest Inc. (Morrisville, PA, USA). NMR (^1^H, ^13^C, HSQC (2D: ^1^H-^13^C)) data of synthesized compounds was collected on Bruker Avance II 600 MHz spectrometer (Billerica, MA, USA) equipped with a BBO room temperature probe. Deuterated dimethyl sulfoxide (DMSO-*d*_6_) containing tetramethylsilane (TMS) was used as a solvent. Mass spectra were collected on Waters Quattro Micro mass spectrometer (Waters Corp., Milford, MA, USA) equipped with an ESI probe and operated in the positive ionization mode. FTIR spectra were collected on a diamond ATR cell attached to Nexus 670 (ThermoFisher, Madison, WI, USA) spectrometer, equipped with a DTGS room temperature detector. Thermogravimetric analysis (TGA) was performed on Q500 TGA (TA instruments, New Castle, DE, USA) at a 10 °C/min heating rate under nitrogen purge (flow rate 60 mL/min). Differential scanning calorimetry (DSC) measurements were performed on a PerkinElmer DSC8500 (Waltham, MA, USA) equipped with a CLN2 liquid nitrogen chiller under a dry helium purge (flow rate 20 mL/min) and a TA Instruments Q1000 equipped with an RCS90 mechanical chiller and operated in T4P mode under a dry nitrogen purge (flow rate 50 mL/min) at heating and cooling rates of 10 °C/min. The instrument temperatures were calibrated against adamantane, benzophenone, indium, tin and lead standards. Heat flow was calibrated against indium and sample temperatures were corrected for the measured heating rate against indium. Samples were sealed in 50 μL cold-welded aluminum hermetic pans in an inert, dry environment prior to measurement. Heat capacity measurements from the Q1000 DSC were taken directly from the instrument software and were not further corrected against a heat capacity standard.

### General Procedure for the Synthesis of Quaternary Ammonium (QA) Monomers

DMAEMA (10.0 mmol) was reacted with alkyl halide (10.0 mmol) at 50 to 55 °C in the presence of BHT (1.0 mmol) and chloroform (2.5 mL). Figure 4 lists all combinations of reacting compounds and their corresponding reaction products. After 24 h, reaction products were collected, purified and dried as indicated below.

*5-Carboxy-N-(2-(methacryloyloxy)ethyl)-N,N-dimethylpentan-1-aminium bromide* (AMadh1), ethanol (2.0 mL) was added to the reaction mixture and transferred to a 50-mL conical separatory flask. Product was precipitated and washed with hexane (3 × 20 mL). The supernatant was discarded and acetone (40 mL) was used to re-dissolve the product. Hexane (10 mL) was added to this solution and AMadh1 precipitated as fine white powder. Product was collected by vacuum filtration, washed with hexane, and vacuum dried. The reaction yielded 2.4250 g of purified product (68.8% yield). FTIR (ATR) ν_max_ 3010.78, 2915.74, 1727.02, 1716.79, 1636.09, 1488.30, 1455.62, 1395.06, 1316.78, 1289.61, 1246.48, 1156.13, 1134.14 946.75 848.19, 814.90, 741.39, 645.12 cm^−1^; ^1^H-NMR (DMSO-*d*_6_, 600 MHz) δ 12.04 (1H, s), 6.08 (1H, s), 5.76 (1H, s), 4.52 (2H, m), 3.70 (2H, m), 3.36 (2H, m), 3.09 (6H, s), 2.24 (2H, t), 1.91 (3H, s), 1.69 (2H, m), 1.55 (2H, m), 1.28 (2H, m); ^13^C-NMR (DMSO-*d*_6_, 151 MHz) δ 174.2, 165.8, 135.3, 126.5, 63.6, 61.6, 58.0, 50.4, 33.2, 25.2, 23.9, 21.5, 17.8; MSEI^+^ *m*/*z* 113.0 (C_6_H_9_O_2_^•^, *m*/*z* = 113.1), 114.1, 172.2 (C_9_H_18_NO_2_^•+^, *m*/*z* = 172.1), 186.3 (C_10_H_20_NO_2_^•+^, *m*/*z* = 186.1), 258.3 (C_13_H_24_NO_4_^2•+^, *m*/*z* = 258.2), 286.3, 300.3.

*10-Carboxy-N-(2-(methacryloyloxy)ethyl)-N,N-dimethyldecan-1-aminium bromide* (AMadh2), the product precipitated out of solution as white powder. AMadh2 was collected by vacuum filtration and washed with chloroform. The reaction yielded 2.0983 g of purified product (53.2% yield). FTIR (ATR) ν_max_ 3020.22, 2919.64, 2853.68, 1720.62, 1632.90, 1472.53, 1450.86, 1396.20, 1321.88, 1298.54, 1205.04, 1160.55, 1102.09, 1029.49, 981.36, 961.54, 911.05, 811.23, 718.91, 649.71 cm^−1^; ^1^H-NMR (DMSO-*d*_6_, 600 MHz) δ 11.96 (1H, s), 6.08 (1H, s), 5.76 (1H, s), 4.52 (2H, m), 3.70 (2H, m), 3.35 (2H, m), 3.09 (6H, s), 2.19 (2H, t), 1.91 (3H, s), 1.67 (2H, m), 1.48 (2H, q), 1.26 (12H, m); ^13^C-NMR (DMSO-*d*_6_, 151 MHz) δ 174.4, 165.8, 135.3, 126.5, 63.8, 61.6, 58.1, 50.4, 33.6, 28.67, 28.64, 28.58, 28.43, 28.37, 25.7, 24.4, 21.7, 17.8; MSEI^+^ *m*/*z* 113.0 (C_6_H_9_O_2_^•^, *m*/*z* = 113.1), 114.2, 342.4, 343.4, 344.4 (C_19_H_36_NO_4_^+^, *m*/*z* = 342.3, 343.3, 344.3).

*N-(2-(methacryloyloxy)ethyl)-N,N-dimethyl-3-(trimethoxysilyl)propan-1-aminium iodide* (AMsil1), chloroform (5.0 mL) was used to rinse and transfer the reaction mixture to a beaker. Diethyl ether (20 mL) was used to precipitate the final product which was collected by vacuum filtration. AMsil1 was washed with hexane and vacuum dried. The reactiosn yielded 4.2420 g of purified product (94.8% yield). FTIR (ATR) ν_max_ 3006.36, 2942.53, 2839.78, 1721.33, 1640.85, 1498.52, 1452.65, 1408.73, 1317.51, 1296.44, 1157.04, 1075.87, 1014.52, 955.81, 930.77, 902.10, 827.52, 808.42, 775.28, 727.63, 654.36 cm^−1^; ^1^H-NMR (DMSO-*d*_6_, 600 MHz) δ 6.09 (1H, s), 5.77 (1H, s), 4.52 (2H, m), 3.70 (2H, m), 3.51 (9H, s), 3.34 (2H, m), 3.09 (6H, s), 1.92 (3H, s), 1.72 (2H, m), 0.54 (2H, t); ^13^C-NMR (DMSO-*d*_6_, 151 MHz) δ 165.8, 135.3, 126.6, 65.9, 61.7, 58.0, 50.6, 50.1, 17.8, 15.6, 5.2; MSEI^+^ *m*/*z* 113.0 (C_6_H_9_O_2_^•^, *m*/*z* = 113.1), 121.0 (C_3_H_9_O_3_Si^•^, *m*/*z* = 121.0), 153.1, 163.2 (C_6_H_15_O_3_Si^•^, *m*/*z* = 163.1), 320.3, 321.3, 322.2 (C_14_H_30_NO_5_Si^+^, *m*/*z* = 320.2, 321.2, 322.2).

*N-(2-(methacryloyloxy)ethyl)-N,N-dimethyl-11-(trimethoxysilyl)undecan-1-aminium bromide* (AMsil2), diethyl ether (10 mL × 3) was added to the reaction mixture to precipitate and wash the product. After each addition of diethyl ether, AMsil2 product was collected by centrifugation (14,000 rpm) and the supernatant top layer decanted. The AMsil2 product was vacuum dried. The reaction yielded 1.8474 g of purified product (36.0% yield). FTIR (ATR) ν_max_ 3401.44, 2924.91, 2853.72, 1720.62, 1637.82, 1465.22, 1318.37, 1295.37, 1159.98, 1090.00, 953.85, 811.57, 655.18, 600.60 cm^−1^; ^1^H-NMR (DMSO-*d*_6_, 600 MHz) δ 6.08 (1H, s), 5.76 (1H, s), 4.52 (2H, m), 3.70 (2H, m), 3.46 (9H, s), 3.36 (2H, m), 3.09 (6H, s), 1.91 (3H, s), 1.67 (2H, m), 1.25 (16H, m), 0.57 (2H, m); ^13^C-NMR (DMSO-*d*_6_, 151 MHz) δ 165.8, 135.3, 126.5, 63.8, 61.6, 58.1, 50.4, 49.9, 32.3, 28.9, 28.8, 28.7, 28.6, 28.4, 25.7, 22.1, 21.7, 17.8, 8.6; MSEI^+^ *m*/*z* 113.0 (C_6_H_9_O_2_^•^, *m*/*z* = 113.1), 114.2, 364.3, 365.4, 366.4 (C_18_H_42_NO_4_Si^•+^, *m*/*z* = 364.3, 365.3, 366.3), 432.4, 433.4, 434.4 (C_22_H_46_NO_5_Si^+^, *m*/*z* = 432.3, 433.3, 434.3).

Due to the well-documented hygroscopic nature of QAs [33,34], all synthesized monomers were stored under vacuum (25 mm Hg).

## 4. Conclusions

Four new polymerizable quaternary ammonium salt monomers with different alkyl chain lengths and functional groups have been successfully synthesized by reacting a tertiary amine (DMAEMA) with various commercially available alkyl halides. Their structures were characterized by FTIR, ^1^H, ^13^C and ^1^H-^13^C HSQC NMR and mass spectrometry and their thermal properties were evaluated. Menschutkin reactions in solvents with relatively low dielectric constant provide a simple method for rapid synthesis and purification of quaternary ammonium salts that may undergo unwanted transformations in more polar or hygroscopic solvents.

## Figures and Tables

**Table 1:** NMR spectral peak assignments of AMadh1 structure.

Position	δ*_C_*	δ_H_
1	126.5	5.76, s; 6.08, s
2	135.3	
3	17.8	1.91, s
4	165.8	
5	58.0	4.52, m
6	61.6	3.70, m
7, 8	50.4	3.09, s
9	63.6	3.36, m
10	21.5	1.69, m
11	25.2	1.28, m
12	23.9	1.55, m
13	33.2	2.24, t
14	174.2	
19		12.04, s

**Table 2:** NMR spectral peak assignments of AMadh2 structure.

Position	δ*_C_*	δ_H_
1	126.5	5.76, s; 6.08, s
2	135.3	
3	17.8	1.91, s
4	165.8	
5	58.1	4.52, m
6	61.6	3.70, m
7, 8	50.4	3.09, s
9	63.8	3.35, m
10	21.7	1.67, m
11–16	25.7, 28.37, 28.43, 28.58, 28.64, 28.67	1.26, m
17	24.4	1.48, q
18	33.6	2.19, t
19	174.4	
20		11.96, s

**Table 3:** NMR spectral peak assignments of AMsil1 structure.

Position	δ*_C_*	δ_H_
1	126.6	5.77, s; 6.09, s
2	135.3	
3	17.8	1.92, s
4	165.8	
5	58.0	4.52, m
6	61.7	3.70, m
7, 8	50.6	3.09, s
9	65.9	3.34, m
10	15.6	1.72, m
11	5.2	0.54, t
12,13,14	50.1	3.51, s

**Table 4:** NMR spectral peak assignments of AMsil2 structure.

Position	δ*_C_*	δ_H_
1	126.5	5.76, s; 6.08, s
2	135.3	
3	17.8	1.91, s
4	165.8	
5	58.1	4.52, m
6	61.6	3.70, m
7, 8	50.4	3.09, s
9	63.8	3.36, m
10	21.7	1.67, m
11–18	22.1, 25.7, 28.4, 28.6, 28.7, 28.8, 28.9, 32.3	1.25, m
19	8.6	0.57, t
20,21,22	49.9	3.46, s

## Supplementary Materials

The supplementary figures are available online.
