# Supplementary material for: Synthesis, Purification and Characterization of Polymerizable Multifunctional Quaternary Ammonium Compounds
Source: Molecules. 2019 Apr 13;24(8):1464. doi: 10.3390/molecules24081464 (PMC6515548; doi:10.3390/molecules24081464)
Supplement: Supplementary file 1 [file molecules-24-01464-s001.pdf]

molecules

Supplementary Information

Synthesis, Purification and Characterization of Polymerizable Multifunctional Quaternary Ammonium Compounds

Ugochukwu C. Okeke [1,*], Chad R. Snyder [2] and Stanislav A. Frukhtbeyn [1,*]

[1] Volpe Research Center, American Dental Association Foundation, 100 Bureau Dr., Stop 8546, Gaithersburg, MD 20899, USA
[2] Materials Science and Engineering Division, National Institute of Standards and Technology, 100 Bureau Dr., Stop 8546, Gaithersburg, MD 20899, USA; chadsnyd@nist.gov
* Corresponding authors: stanislav.frukhtbeyn@nist.gov (S.A.F.); okekeugo@gmail.com (U.C.O.); Tel.: +301-975-6808 (S.A.F.); +301-975-5861 (U.C.O.); Fax: +301-963-9143 (S.A.F. & U.C.O.)

Contents:

Figure S1. FTIR spectra of AMadh1 (top) and AMadh2 (bottom).

Figure S2. FTIR spectra of AMsil1 (top) and AMsil2 (bottom).

Figure S3. ^{1}H-NMR spectra of DMAEMA.

Figure S4. ^{13}C-NMR spectra of AMadh1 (top) and AMadh2 (bottom).

Figure S5. ^{13}C-NMR spectra of AMsil1 (top) and AMsil2 (bottom).

Figure S6. ^{1}H(F2)-^{13}C(F1) HSQC NMR spectra of AMadh1.

Figure S7. ^{1}H(F2)-^{13}C(F1) HSQC NMR spectra of AMadh2.

Figure S8. ^{1}H(F2)-^{13}C(F1) HSQC NMR spectra of AMsil1.

Figure S9. ^{1}H(F2)-^{13}C(F1) HSQC NMR spectra of AMsil2.

Figure S10. Mass spectra of AMadh1.

Figure S11. Mass spectra of AMadh2.

Figure S12. Mass spectra of AMsil1.

Figure S13. Mass spectra of AMsil2.

Figure S14. Typical TGA thermograms of AMadh1, AMadh2, AMsil1, and AMsil2.

Figure S15. Typical first heating, first cooling, and second heating DSC scans (10 °C/min) of AMadh1.

Figure S16. Typical first heating, first cooling, and second heating DSC scans (10 °C/min) of AMadh2.

Figure S17. Typical first heating, first cooling, and second heating DSC scans (10 °C/min) of AMsil1.

Figure S18. Typical first heating, first cooling, and second heating DSC scans (10 °C/min) of AMsil2.

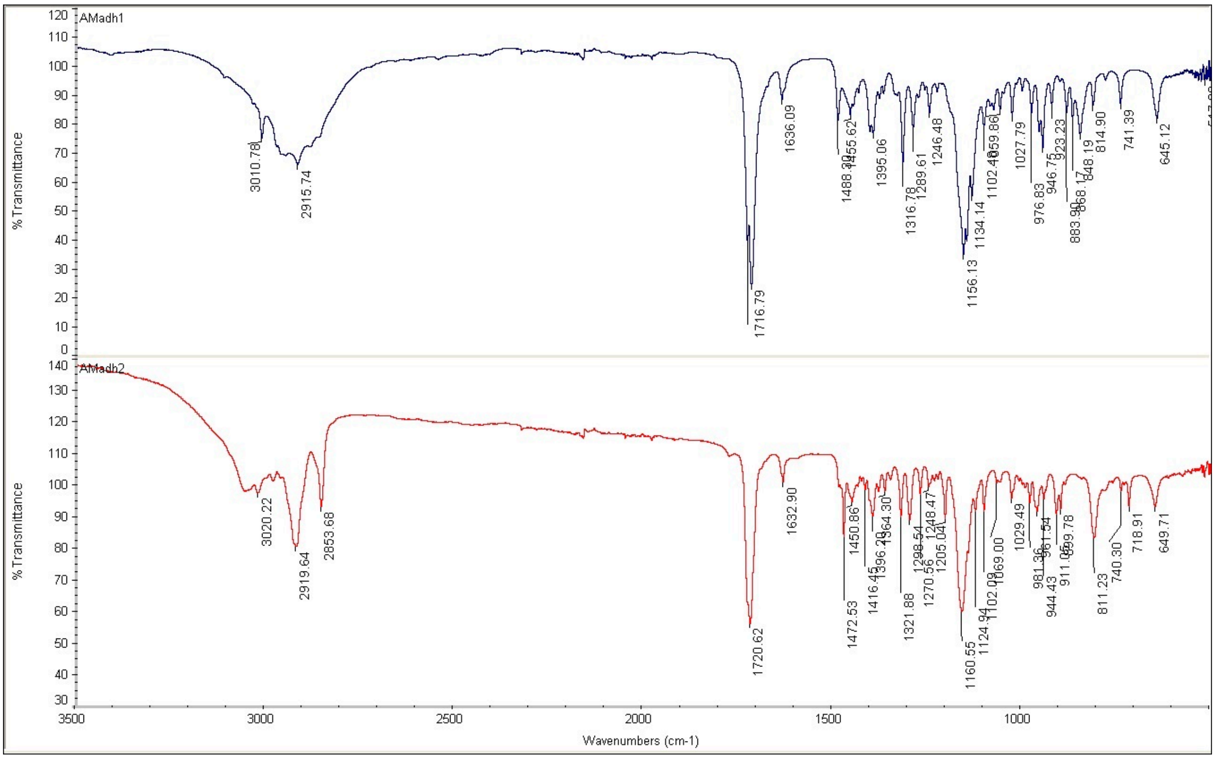

Figure S1. FTIR spectra of AMadh1 (**top**) and AMadh2 (**bottom**).

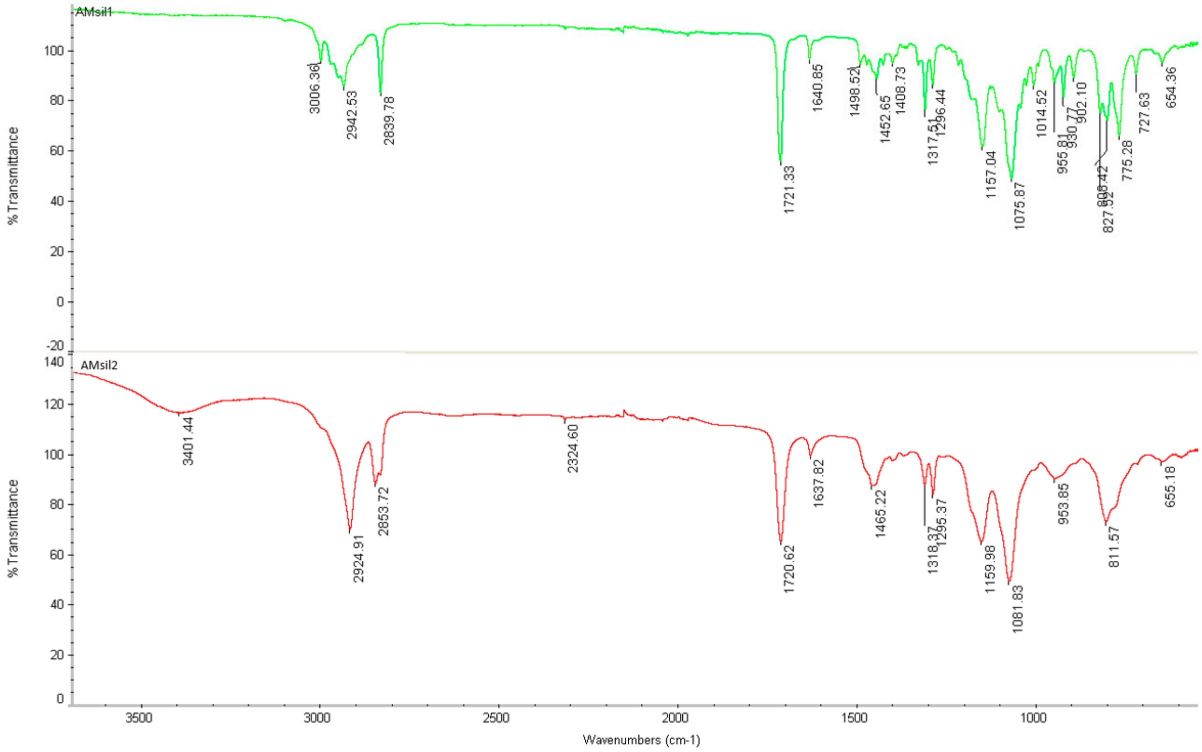

Figure S2. FTIR spectra of AMsil1 (**top**) and AMsil2 (**bottom**).

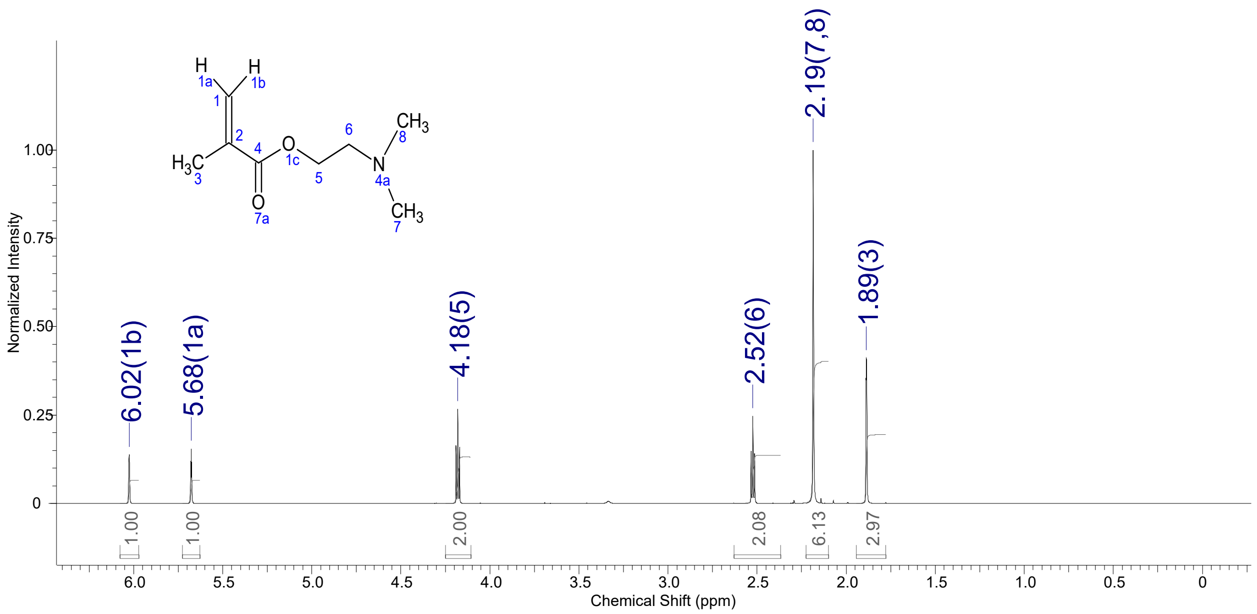

Figure S3. ^{1}H-NMR spectra of DMAEMA.

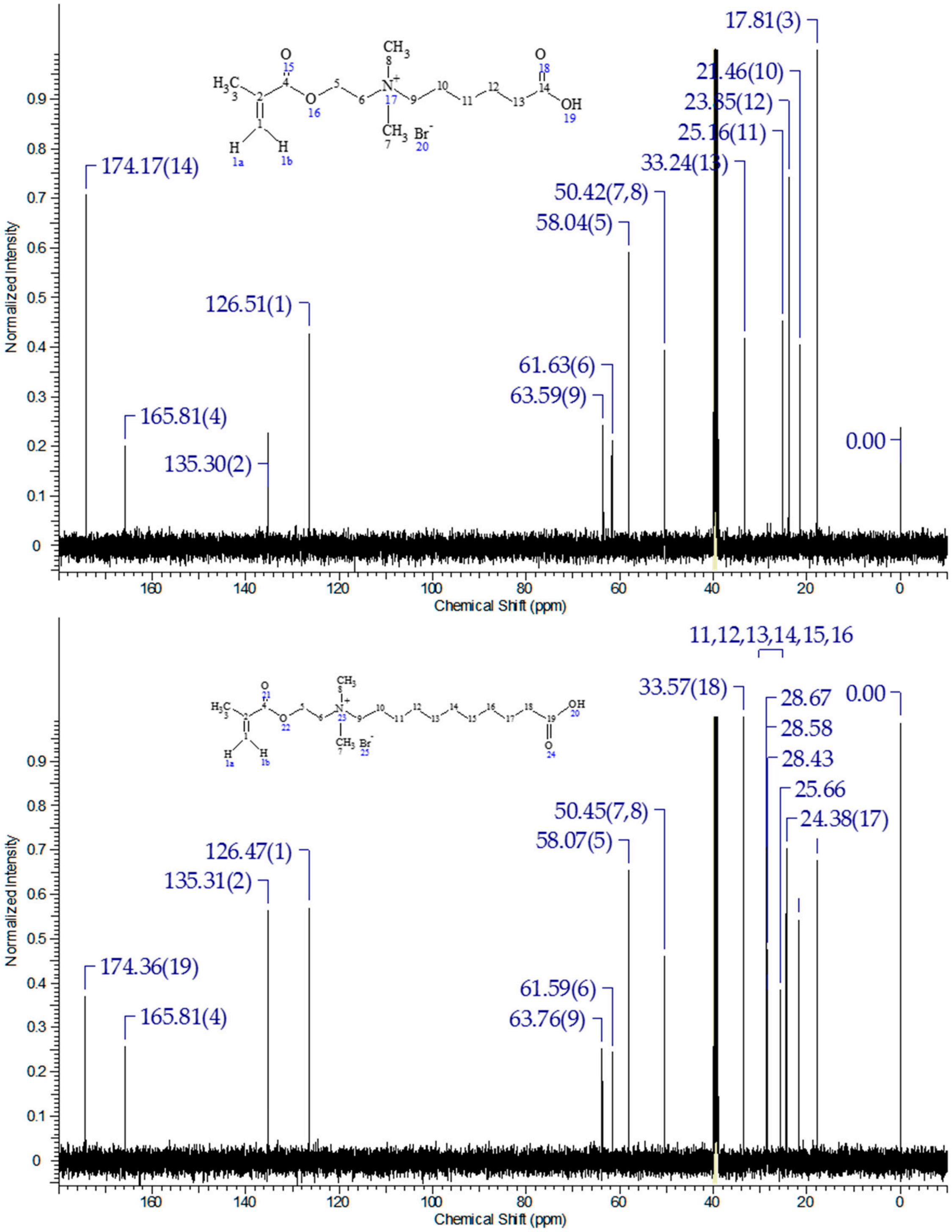

Figure S4. ^{13}C-NMR spectra of AMadh1 (**top**) and AMadh2 (**bottom**).

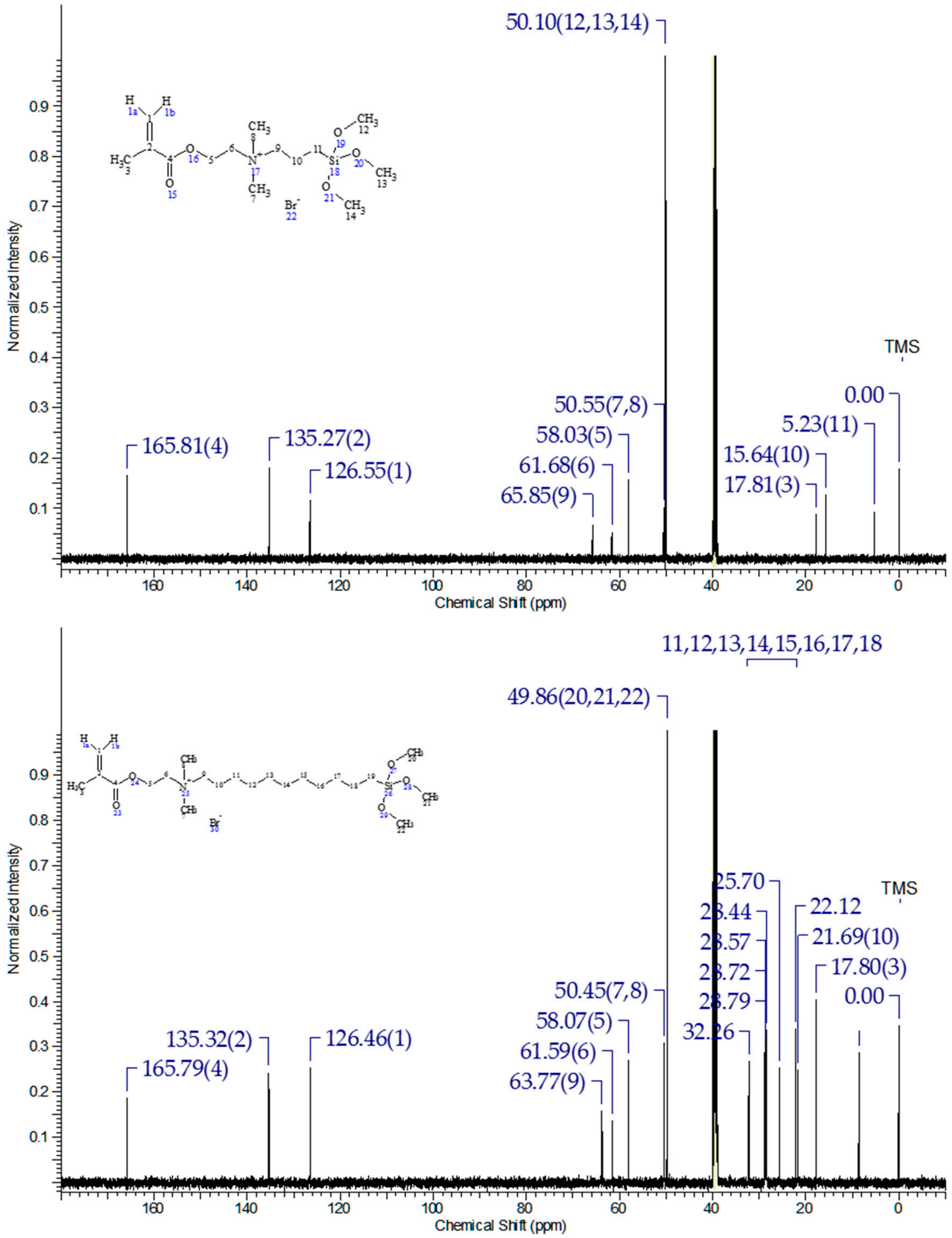

Figure S5. ^{13}C-NMR spectra of AMsil1 (top) and AMsil2 (bottom).

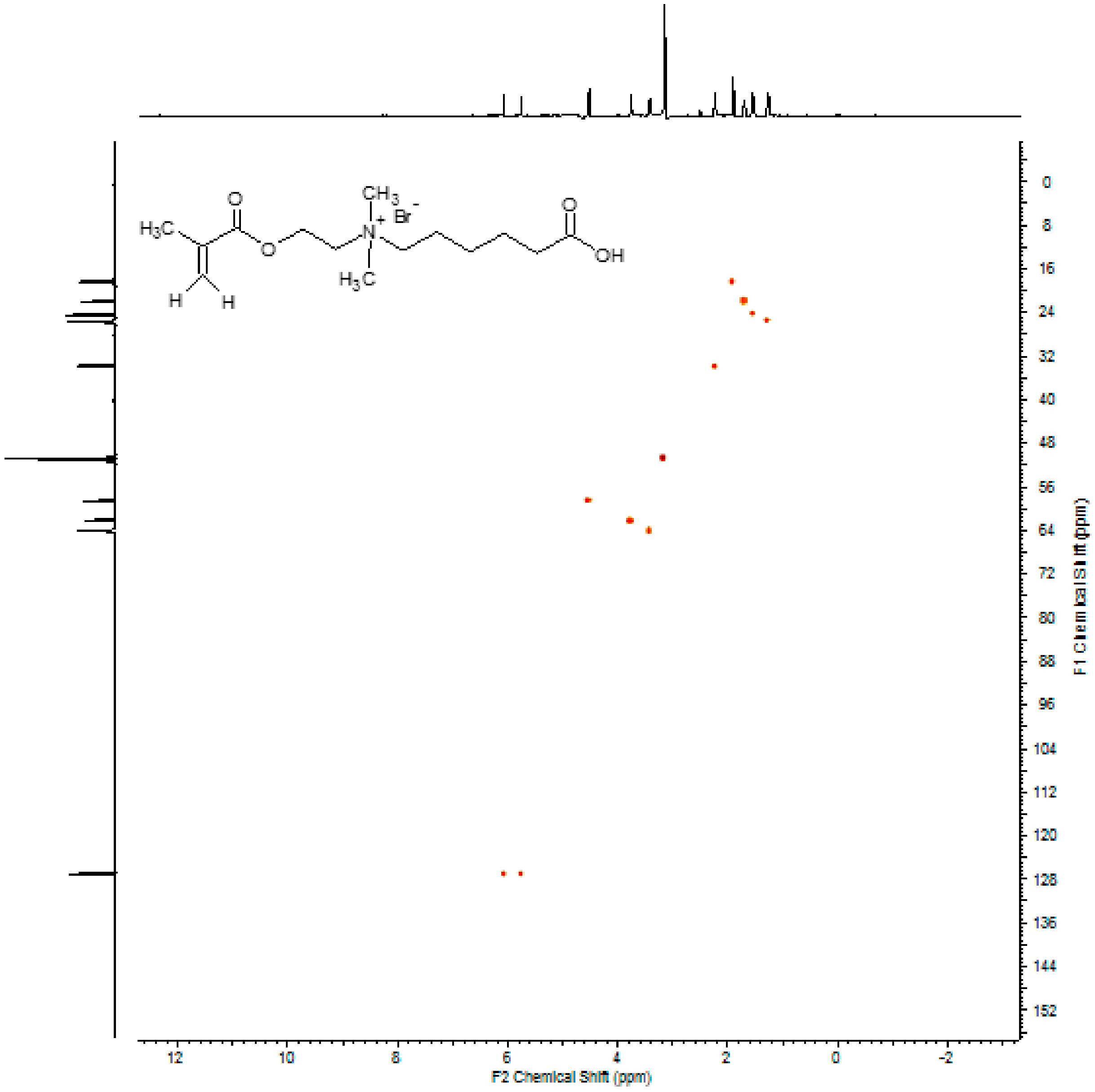

Figure 6. ^{1}H(F2)-^{13}C(F1) HSQC NMR spectra of AMadh1.

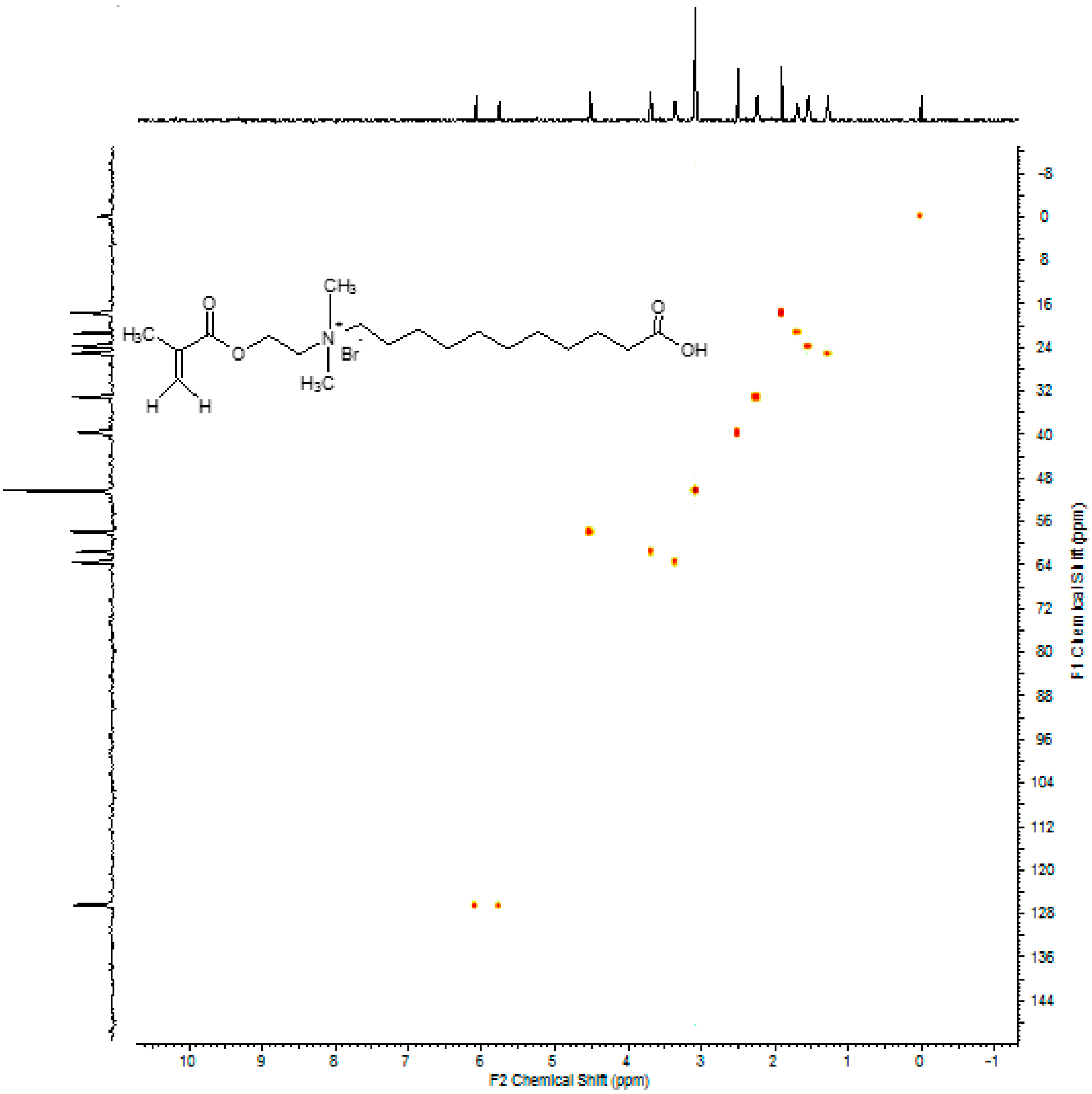

Figure S7. $^{1}H(F2)$-$^{13}C(F1)$ HSQC NMR spectra of AMadh2.

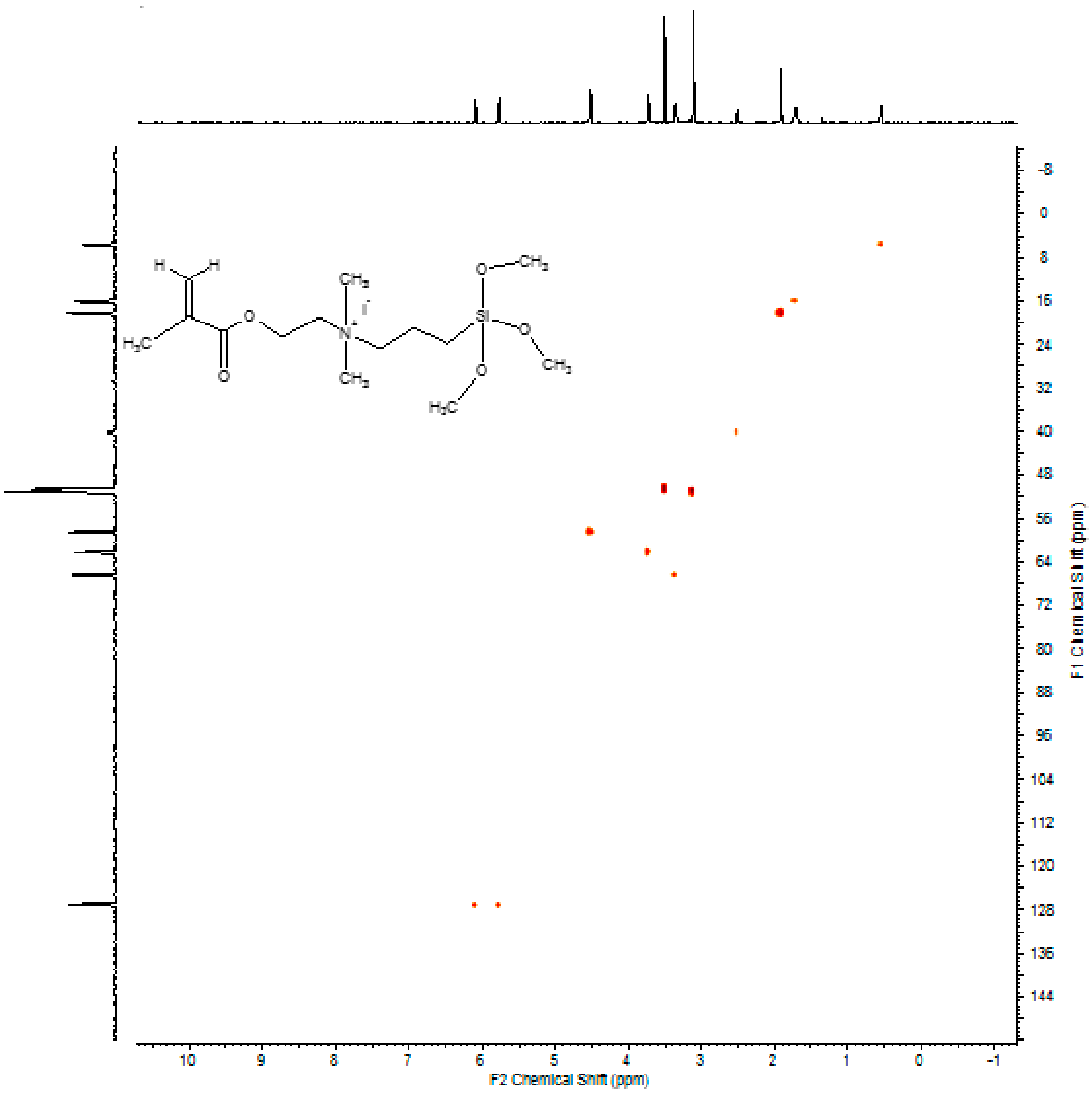

Figure S8. $^{1}H(F2)-^{13}C(F1)$ HSQC NMR spectra of AMsil1.

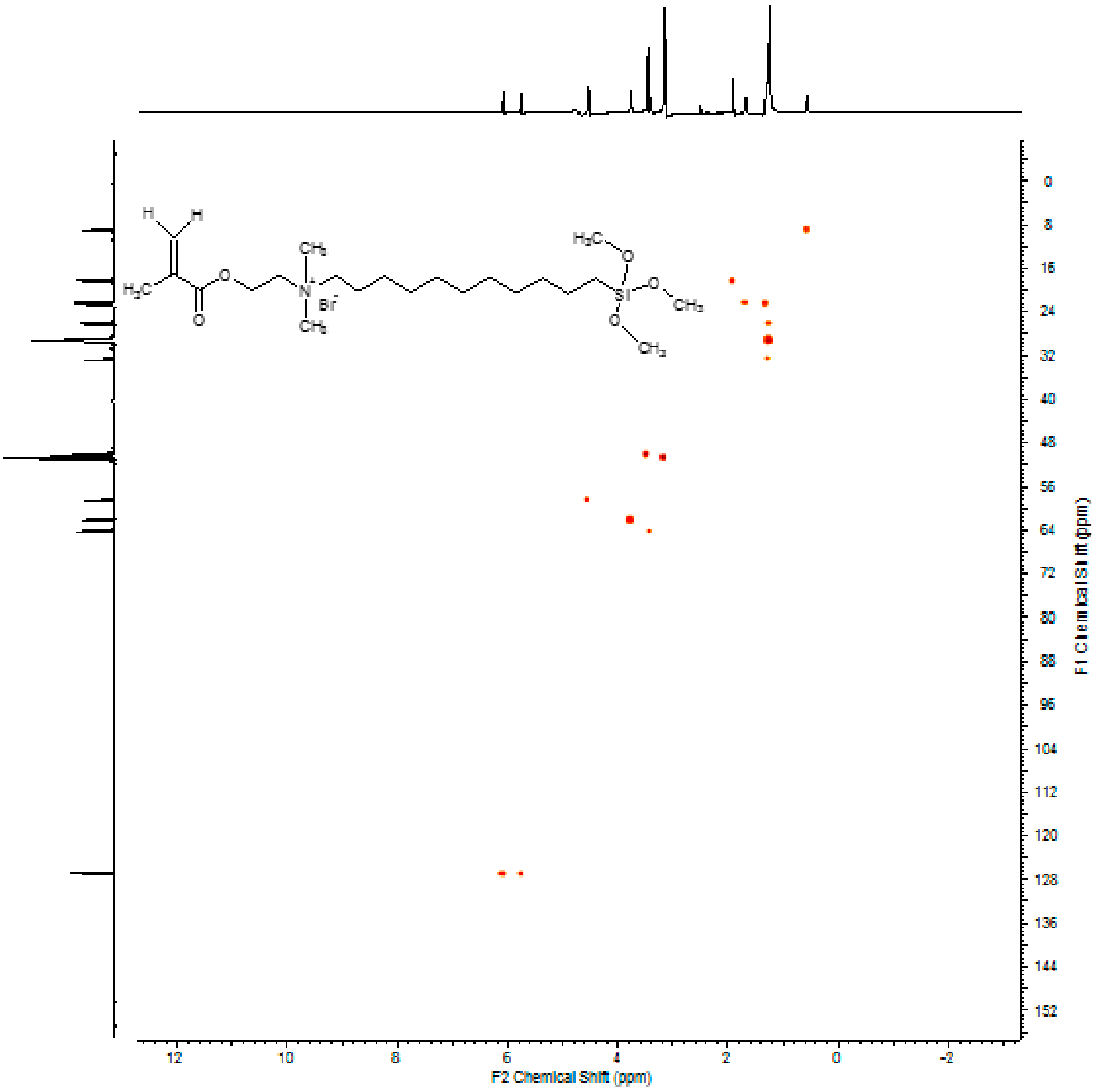

Figure S9. ^{1}H(F2)-^{13}C(F1) HSQC NMR spectra of AMsil2.

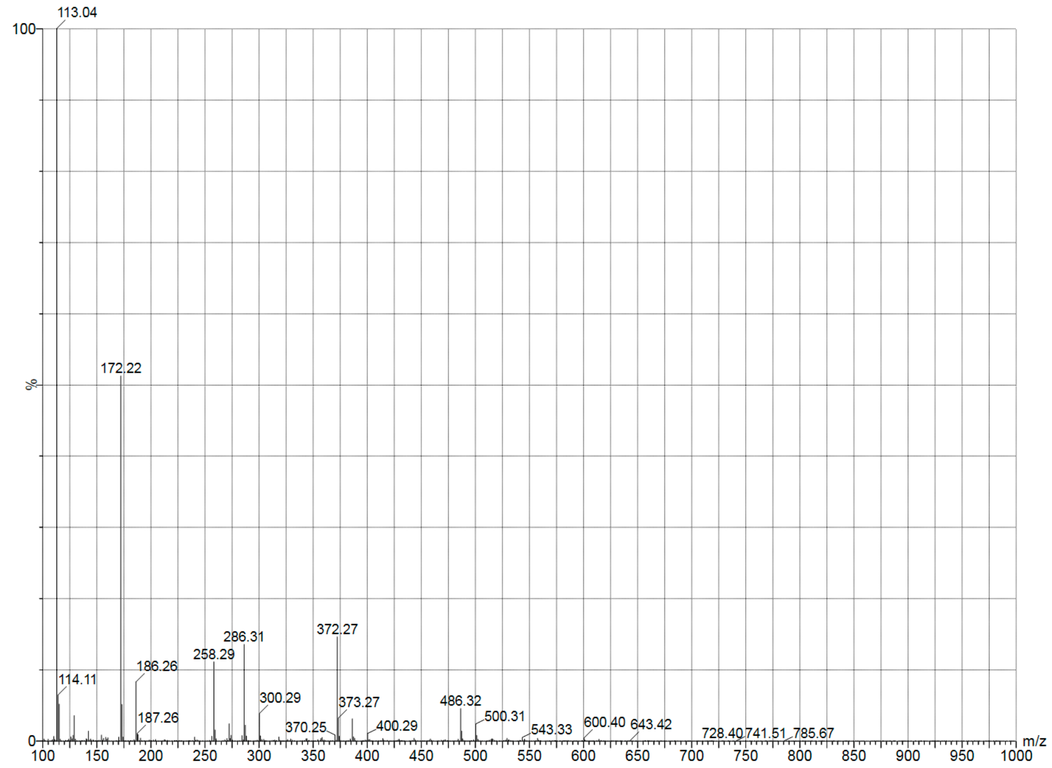

Figure S10. Mass spectra of AMadh1.

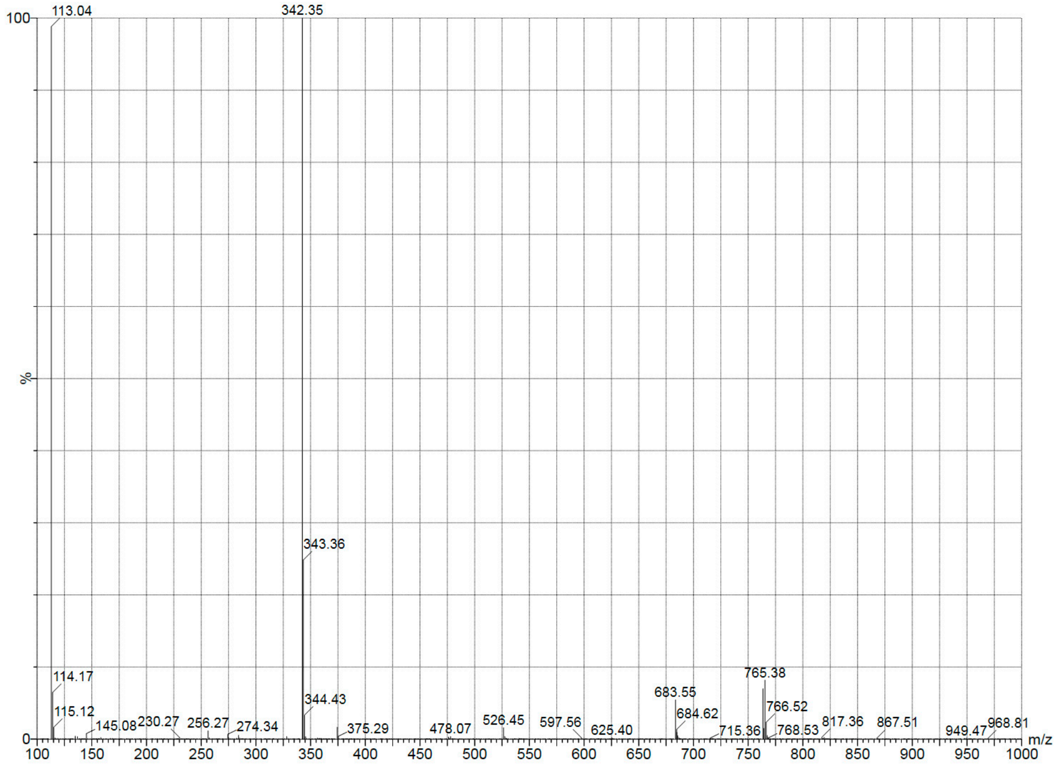

Figure S11. Mass spectra of AMadh2.

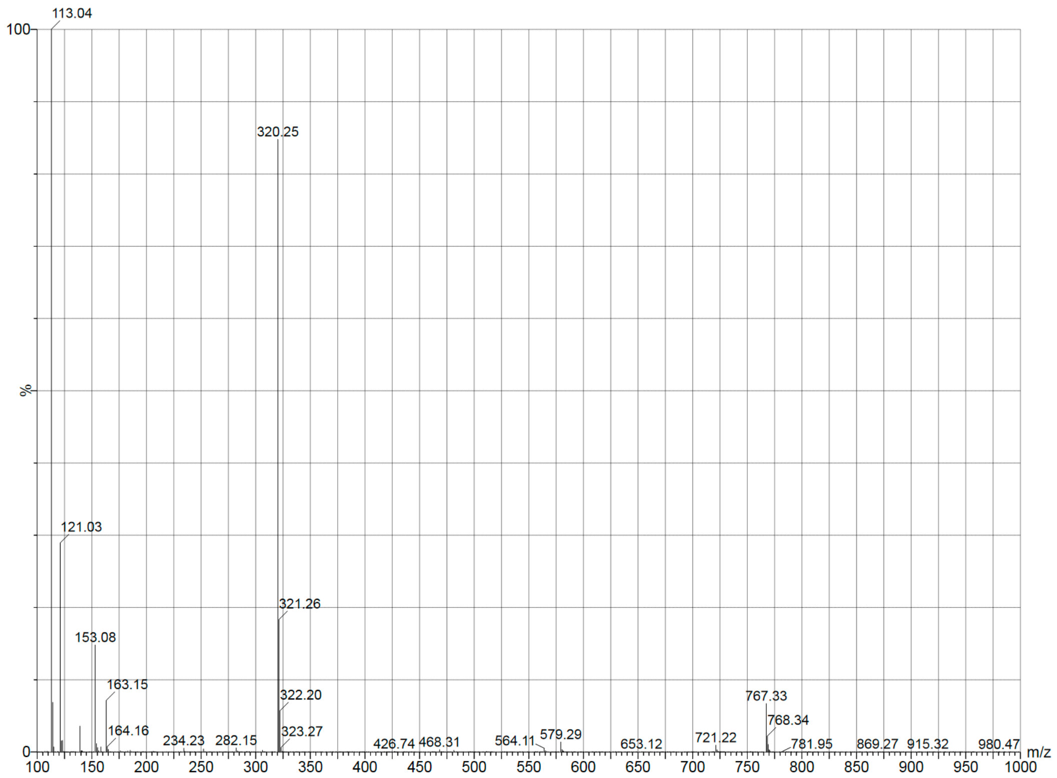

Figure S12. Mass spectra of AMsil1.

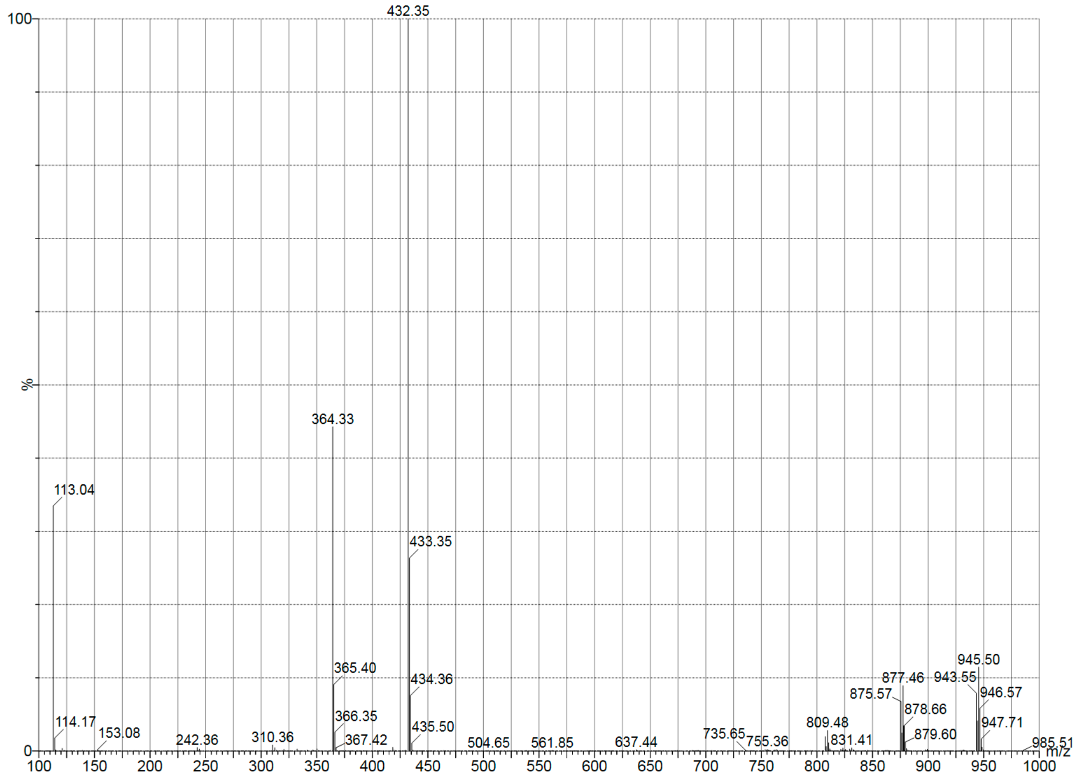

Figure S13. Mass spectra of AMsil2.

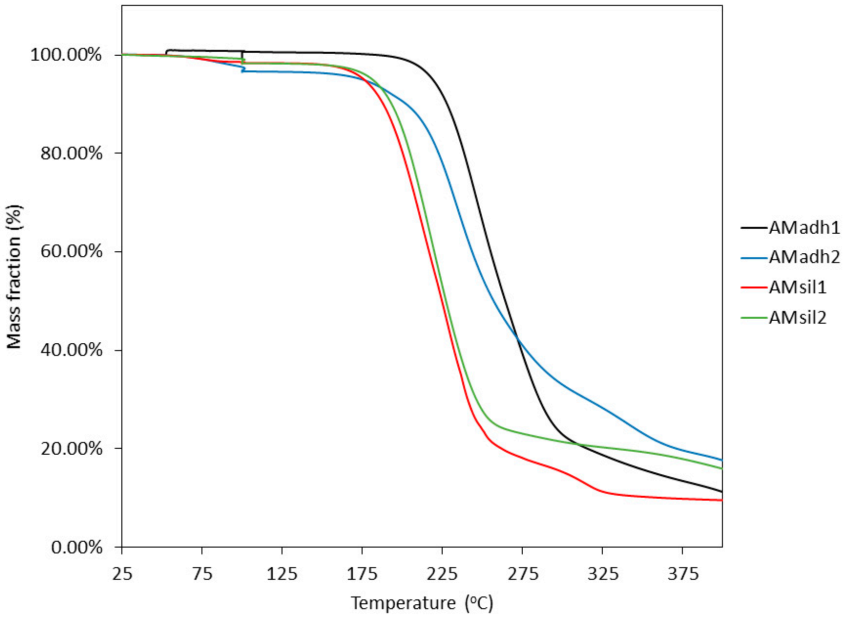

Figure S14. Typical TGA thermograms of AMadh1, AMadh2, AMsil1, and AMsil2.

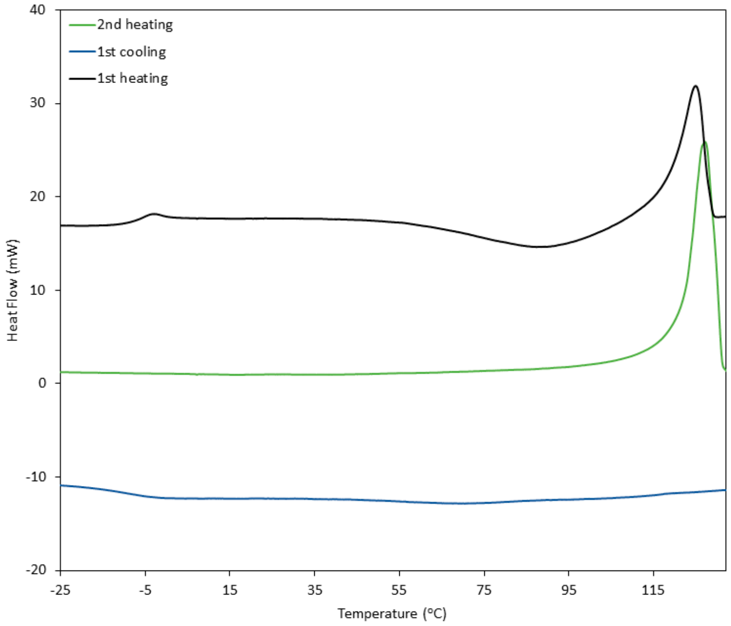

Figure S15. Typical first heating, first cooling, and second heating DSC scans (10 °C/min) of AMadh1.

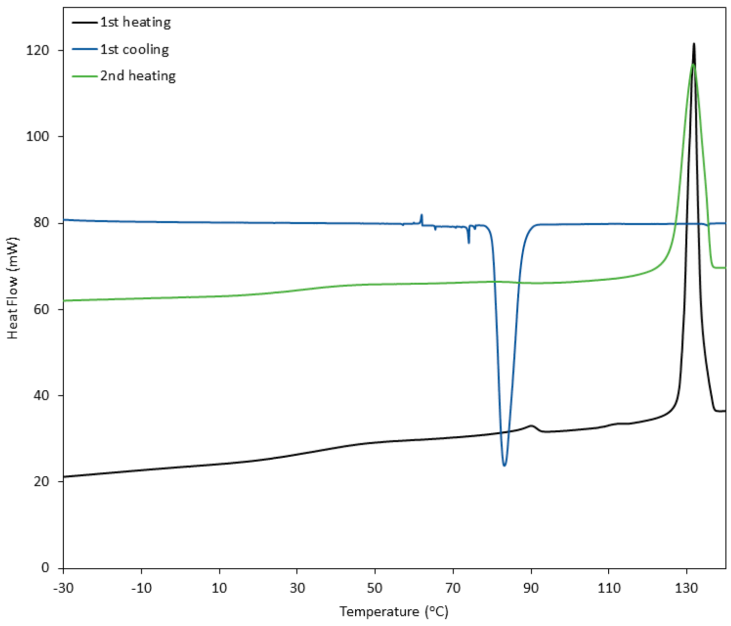

Figure S16. Typical first heating, first cooling, and second heating DSC scans (10 °C/min) of AMadh2.

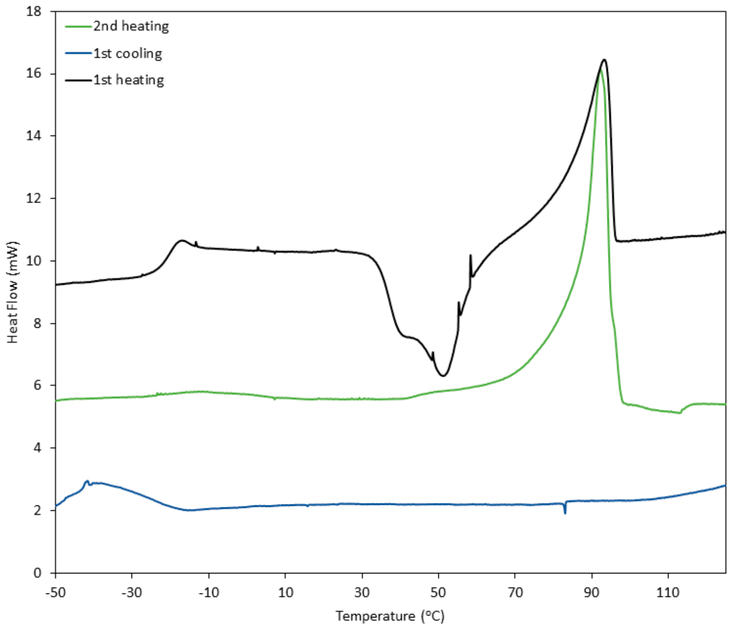

Figure S17. Typical first heating, first cooling, and second heating DSC scans (10 °C/min) of AMsil1.

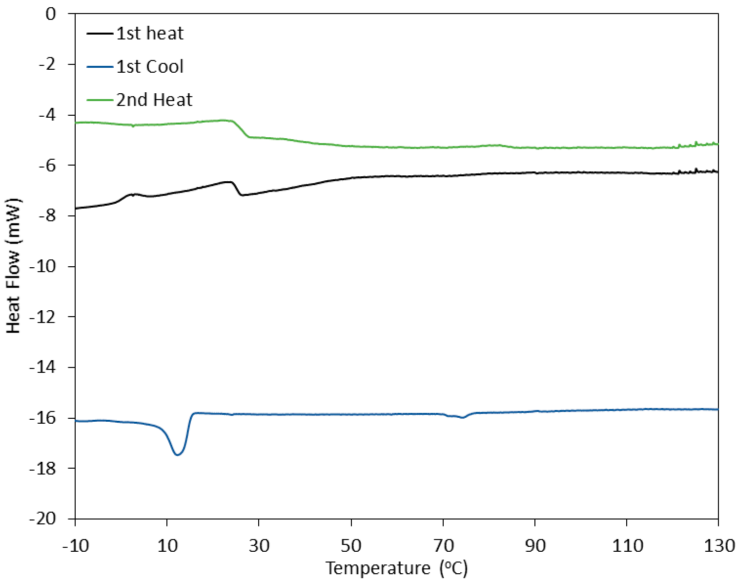

Figure S18. Typical first heating, first cooling, and second heating DSC scans (10 °C/min) of AMsil2.
